# Study of polymethylmethacrylate/tricalcium silicate composite cement for orthopedic application

**DOI:** 10.1016/j.bj.2022.05.005

**Published:** 2022-05-29

**Authors:** Yang Wei, Nareshkumar Baskaran, Huey-Yuan Wang, Yu-Chieh Su, Sasza Chyntara Nabilla, Ren-Jei Chung

**Affiliations:** a Department of Chemical Engineering and Biotechnology, National Taipei University of Technology (Taipei Tech), Taipei, Taiwan; b Department of Stomatology, MacKay Memorial Hospital, Taipei, Taiwan; c Department of Materials, University of Oxford, Oxford, United Kingdom

**Keywords:** Tricalcium silicate, Bone cement, Polymethylmethacrylate, Organic-inorganic composite

## Abstract

**Background:**

Among orthopedic surgery materials, poly (methyl methacrylate) (PMMA) is most commonly used for its excellent mechanical properties and rapid self-setting time. However, PMMA bone cement has been reported to cause thermal necrosis and to have poor bioactivity, which must be improved. In contrast, tricalcium silicate (TCS), the most significant component of Portland Cement and the most effective bone cement material, might not always meet the needs of the cement due to its poor mechanical properties and elevated pH levels during hydration. We hypothesize that the benefits of both PMMA and TCS can be harnessed by mixing them together in different proportions. This would represent a better solution for the issues faced when using them alone.

**Methods:**

We, therefore, prepared a novel organic-inorganic PMMA/TCS composite bone cement mixing PMMA and different amounts of TCS and tested its effect on the biophysical properties.

**Results:**

The addition of 30% TCS reduced the exothermic temperature and pH variation during cement setting and hydration processes. However, the mechanical and handling properties of the bioactive PMMA/TCS composite were not affected. The *in vitro* study also revealed that the composite materials had higher cell viability than pure PMMA and TCS. Also, the *in vivo* study on animals indicated that the composite materials were more capable of forming bone, which further reinforced the biocompatibility of the proposed PMMA/TCS bone cement.

**Conclusion:**

By combining the advantages of each component, it could be possible to construct a more effective composite bone cement material. This would meet the needs of implantation material for orthopedic surgeries or a possible bone filler.

## Graphical abstract

**Figure:**
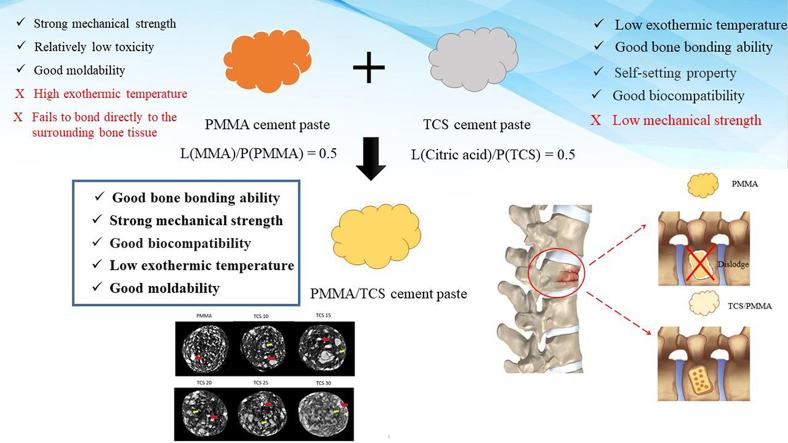

**At a glance commentary**

**Scientific background on the subject**

Poly (methylmethacrylate) (PMMA) is widely used for implant fixation in orthopedic and trauma surgery. However, it suffers from high exothermic reaction temperatures, low bioactivity, and monomer toxicity. Combining PMMA with inorganic fillers has shown improved properties. This study used tricalcium silicate (TCS) as an inorganic filler to develop a novel PMMA/TCS bone cement composite.

**What this study adds to the field**

Our study results suggest that the addition of TCS in the composite within the selected concentration range could induce bone regeneration and stabilize pH and exothermic temperature without compromising the mechanical and handling characteristics of PMMA bone cement. This could be a potential bone filler for various orthopedic surgeries.

## Background

A bone tissue substitute is typically used to guide bone regeneration, leading to the development of artificial bone tissue substitutes such as bone cement. Clinically, the injection of bone cement using minimally invasive techniques has significant potential due to its advantages such as minimal secondary damage, flexibility in the operation process, and shorter hospital stays [1].

In bone cement development, poly (methylmethacrylate) (PMMA)-based cement or its derivatives have been widely used for over 50 years to stabilize or fix the artificial joints or implants at the defect sites such as hip, wrist, shoulder, elbow and knee during various orthopedic and trauma surgeries [2,3]. PMMA cement has superior self-setting properties. As a result of reacting with radicals [4], PMMA powder and methyl methacrylate monomer form a long chain polymer to fix the defect [5]. The resulting polymers form a firm mechanical bond with implants by distributing implant loads evenly [1]. While using PMMA bone cement, some of the major problems encountered are high exothermic reaction temperatures, low bioactivity, and monomer toxicity [4,[6], [7], [8]]. Additionally, PMMA joint replacements have often failed because of the complications mentioned above, which result in aseptic loosening and tissue necrosis [[9], [10], [11]].

As an alternative, inorganic cement with the significant components of calcium phosphate and calcium silicate, which have similar compositions to natural bones, was initially used to replace PMMA in the reconstruction of damaged bones [12]. Through a non-exothermic process, the inorganic bone cement could be hydrated and set to compact solids [13]. Finally, the degraded material in the defect would be released and absorbed safely [13,14].

Inorganic cement may have a limited mechanical property, particularly in terms of bending resistance and brittleness [15]. Additionally, the calcium hydroxide (Ca(OH)_2_) that forms during the hydration process of inorganic cement is likely to raise the pH of surrounding tissues [16,17]. Consequently, composite cement was developed by combining inorganic ceramic with a polymer to improve the bioactivity of PMMA bone cement and to enhance its mechanical properties by strengthening the bone-cement interface while reducing the precipitation of Ca(OH)_2_ [8,18]. Unlike PMMA bone cement alone, which is poorly compatible with adjacent bone tissue, composite PMMA with bioactive inorganic ingredients may react in various ways with living tissue [[19], [20], [21], [22]].

Among the inorganic fillers to PMMA bone cement, Portland Cement (P.C.) was considered an economical and viable alternative to other inorganic ingredients. P.C.-based materials have been used successfully for composite PMMA cement bioactivation [23]. However, Portland cement is a combination of four major components, including dicalcium silicate (C_2_S), tricalcium silicate (C_3_S, TCS), tricalcium aluminate (C_3_A), and tetra calcium aluminoferrite (C_4_AF). Preparing this complicated composition is tedious and leads to diverse bioactive properties due to its various origins and preparation types [24]. Therefore, we proposed that we use more purified P.C. content in the PMMA bone cement, which would result in a more physicochemically characterized composite cement. For example, TCS, which is about 68% of the total P.C. components, has higher calcium and oxide ion content in the lattice, making it more bioactive than other P.C. components [25,26].

This project aimed to develop a novel PMMA/TCS bone cement composite combining the desired properties of PMMA and TCS. The design of PMMA bone cement combined with TCS ceramics has not yet been described. The effect of TCS on PMMA bone cement's physicochemical and biological properties was studied systematically. Innovative composite bone cement was then developed based on the required properties of each component, such as a modified PMMA bone cement combining PMMA and TCS.

## Experimental section

### PMMA/TCS bone cement preparation

We have applied two steps to prepare bone cement specimens. The detailed information on the preparation of raw materials for the bone cement can be seen in the supplementary section S1. Initially, a given solid component and the liquid PMMA monomer were mixed in a vibrator for 6 h under ambient conditions (relative humidity: ≥40%; temperature: 24 °C). After that, previously prepared TCS powder was mixed with the chosen optimized concentration of 0.5 M citric acid solution to accelerate the hydration of the mixtures (See the section S5 in the supplementary data) with a ratio of 2:1. The TCS powder/citric acid was thoroughly blended with PMMA bone cement paste in different weight ratios ranging from 10, 15, 20, 25, 30, 40, 60–80%, denoted as TCS 10, 15, 20, 25, 30, 40, 60 and 80, respectively.

### Handling characteristics (working and setting times)

The working and setting time was measured using Gillmore needles [27,28]. TCS powder then mixed with PMMA in paste form in different ratios and placed into poly (tetrafluoroethylene) (PTFE) molds (10 mm diameter and 6 mm height) for a given period. Working time represents the available time from manipulating the cement from the initial mixture until a semihard stage under a 0.3 MPa static pressure. In addition, the setting time defines as the corresponding time for a static load of 5 MPa [29].

### Compression test

According to ASTM, the complete set column specimens (diameter = 6 mm, height = 12 mm) was measured with Instron-1195 (USA), at a loading strain rate of 0.5 mm min^−1^ D695-91 to obtain the compressive strength. In this study, the compression was measured at a 20 mm/min speed until failure under room temperature. The compressive strength (MPa) was obtained from evaluating maximum stress after soaking in the deionized (DI) water for 7 and 14 days.

### Characterization of the PMMA/TCS bone cement specimens

#### XRD analysis

The preliminary biocompatibility of material was evaluated through SBF immersion as prepared following supplementary section S2. After soaking the PMMA/TCS bone cement composites in SBF for seven days, the samples were dried and grounded in a mortar. The crystalline structure of the powdered samples was characterized using X-ray diffraction (XRD; Geigerflex, Rigaku, Japan). The attached detector with Cu-Kα radiation (*λ* = 0.15406 nm) operating at 40 mA and 40 kV. The diffraction data were collected in the range of 10–70° (2θ).

#### Morphology observation

Before and after soaking in SBF, the morphology differences in the PMMA/TCS bone cement samples were observed using a field-emission scanning electron microscope (JSM-7610 F, JEOL Ltd., Tokyo, Japan) at a potential of 20 kV. In addition, we have all samples prepared to be conductive by sputter-coating with platinum for morphological analysis.

#### pH measurement

For the pH measurement, groups of specimens were shaped into 10 mm in diameter and 5 mm in height [1] in PTFE molds. The samples were soaked in SBF (pH = 7.4) at 37 °C for weeks. The suspension was collected, and the pH was measured every day for 21 days. The pH variation can be evaluated *in vitro* by immersion studies in SBF solution.

### Measurement of exothermic temperature

We have the exothermic temperature–time profiles during the solidification of specimens evaluated with a K-type thermocouple (Thermo sensors corporation, Garland, TX) connected to a temperature controller (Camsco Electronics Co. Ltd., China). PMMA/TCS bone cement samples with different weight ratios of TCS were molded in a PTFE mold (height = 10 mm, diameter = 5 mm). The temperatures were measured and noted every 30 s for 15 min at 24 °C. The peak temperature (*T*_max_) indicates the highest temperature reached from the detectable maximum exothermic in the polymerization reaction [30].

### Micro-computed tomography (micro CT) analysis

The distribution of TCS additives in the PMMA matrix was verified using the micro-computed tomography technique (Micro-CT). PMMA/TCS bone cement with different TCS content was examined using a desk-top Micro-CT imager (SkyScan 1172, Belgium). The system was operated at an image resolution of 35 μm voxel with current and voltage maintained at 100 μA and 80 kV, respectively, and a 1.0 mm aluminum filter.

### *In vitro* study

#### Cell viability assay and alkaline phosphatase activity assay (ALP)

We have the osteoblast-like MG-63 cell line from human osteosarcoma (ATCC CRL-1427, American Tissue Culture Collection, Rockville, USA) applied to analyze the various PMMA/TCS bone cement combinations. Dulbecco's Modified Eagle Medium (DMEM) with 10% fetal bovine serum (FBS) and 1% penicillin-streptomycin (P.S.) solution were purchased from Sigma–Aldrich (USA), and were used as the complete growth culture medium. The MG-63 cells were cultured in the growth culture medium at 37 °C and maintained in a 5% CO_2_ incubator.

A well-known MTT (3-(4,5-Dimethylthiazol-2-yl)-2,5-Diphenyltetrazolium Bromide, Sigma) colorimetric assay was used to evaluate the cell viability of the PMMA/TCS bone cement samples with different weight ratios of TCS. This test was conducted following ISO 10993–5 [31]. The detailed procedure of this test has been provided in the section S3 in the supplementary data.

Considering the presence of alkaline phosphate (ALP) activity, the ALP T1016 kit (ThermoFisher Scientific, Springfield, USA) was equipped to determine the ALP production, a marker of osteogenesis. Following an early seeding of MG-63 cells (5 × 10^4^) on the bone composites surface, we have the test conducted at 1, 3, 7, 14, and 21 days, accompanied by a culture medium changing thrice per week. Finally, we have the ALP production on the composite surfaces quantified with an ELISA reader at 405 nm.

### Preliminary *in vivo* study in *animals* and histological analysis

To understand the *in vivo* biocompatibility of the materials, preliminary animal studies were performed on rat calvarial defects model under the Guide for the Care, and Use of Laboratory Animal approved by the National Taipei University of Technology (Taipei, Taiwan) to evaluate the biocompatibility of the PMMA/TCS composite (i.e., TCS 30). Entire procedures were done at MacKay Memorial Hospital (Taipei, Taiwan) with prior review and approval of the Institutional Animal Care and Use Committee (IACUC) (MMH-A-S-106-18). Experimental procedures were entirely performed under aseptic conditions to prevent the infection. We have two randomly selected groups with five rats in each one: (i) the control one was the blank group without any implantation, and (ii) the test group was the PMMA/TCS samples with TCS 30 implantation. The complete procedure can be seen in the section S4 in the supplementary data. Following the hematoxylin-eosin (HE) staining, the histological sections were analyzed using a Nikon Eclipse 50i upright microscope (Nikon, Japan). Three wounds per group were conducted.

### Statistical analysis

All the obtained data from the handling, compression, and *in vitro* experiments were repeated five times (n = 5), and the reuslts were represented as mean ± standard deviation. At a *p-*value <0.05, the obtained data with the Student's t-test were regarded as statistically significant.

## Results and discussion

The material must fulfil all the required characteristics that the bone cement should have. However, none of them is ideal, and there is still some limitation. In which case, we propose to have a bioactive bone cement composite prepared with TCS as additives and mixed with conventional PMMA to integrate the lowered cost and excellent bioactivity benefits of TCS with the high strength and fast setting properties of the pure PMMA. Therefore, we have the following results and discussions to investigate the PMMA/TCS composite characteristics, including their *in vitro* bioactivity.

### Optimal TCS concentrations determined from setting times and compressive strength of composite cement

One of cement's most critical handling characteristics is its working and setting time, varied when their composition, mixing environment, and the presented additives were changed [27]. In the clinical trial application, the composite bone cement needed to inject immediately before the final working and setting time, and the wound was closed. The primary reason for the quick setting and strength of the bone cement is its hydration product in a solid network [32]. We have the 0.5 M citric acid mixed with TCS to accelerate the hydration of the mixtures due to the rapid acid–base reaction involved [33]. Figure S1 was provided to illustrate that citric acid could enhance the hydration of the mixtures.

**Fig. 1:**
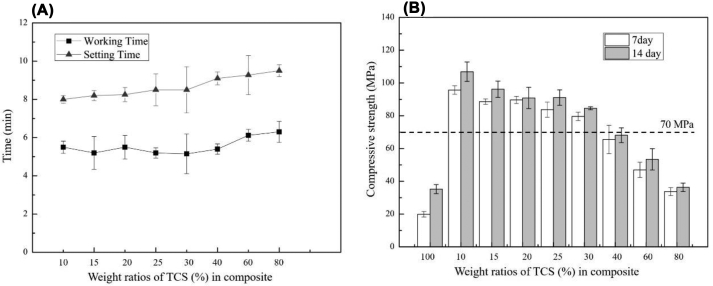
(A)The setting and working times and the (B) compressive strength of PMMA/TCS cements with different weight ratios of TCS. Error bars represent the averaged values ± SD, *n* = 5.

As shown in Fig. 1(A), we can clearly see that there are no significant difference in the working and setting time when the compositions of TCS in the PMMA/TCS composite were increased from 10% to 80%. However, we can also see that there has been a significant difference in the setting time when we compare TCS 10 with TCS 80, possibly due to the relatively slow hydration of TCS. Nevertheless, with the addition of citric acid, all cement samples under the test had setting time within the range of 8–10 min, which still met the clinical demands. The optimal setting time required is between 10 and 15 min [34].

The compressive strength change in the PMMA/TCS bone cement with different weight ratios of TCS (10%–80%) after 7 and 14 days of setting is represented in the Fig. 1(B). It showed that the compressive strength of 100% TCS cement was decreased when compared with all the composites of PMMA/TCS (*p* < 0.05) except the TCS 80, which showed similar compressive strength. The composite with 10% TCS produced a maximum strength value. However, the value was significantly reduced when the TCS composition was increased. The diminution phenomenon observed might be due to the discontinuous and disintegrated PMMA matrix after the addition of TCS, with the whole structure becoming porous, which causes a decline in compressive strength [33]. A similar trend of the compressive strength change of PMMA/TCS with different weight ratios of TCS was observed after 7 and 14 days, which implies no apparent dissolution of the cement during this period [35].

The minimum required compressive strength value of 70 MPa was defined in ISO-5833-2002 for the cured bone cement [36], and all commercially available bone cement must satisfy this standard. Therefore, high concentrations of TCS in PMMA might lead to detrimental mechanical properties, whereas TCS 40 to TCS 80 were not following ISO-5833-2002. However, the compressive strength values of 10–30% TCS were still above 70 MPa. Furthermore, the compressive strength of TCS 10 reached 108 MPa after curing for 14 days, which is comparably much higher than both pure PMMA cement and other combinations [37]. Based on these results, the composite cement's microstructure is considered crucial for its mechanical strength, and it could be adjusted by varying the weight ratio of TCS and PMMA. Therefore, we have the optimal ratio of TCS selected lower than 30% in terms of the required compressive strength. This optimized weight ratio of TCS (10%–30%) will be applied to blend with PMMA to prepare the composite bone cement for further investigations.

### Characterizations of PMMA/TCS bone cement with selected TCS ratios

**Fig. 2:**
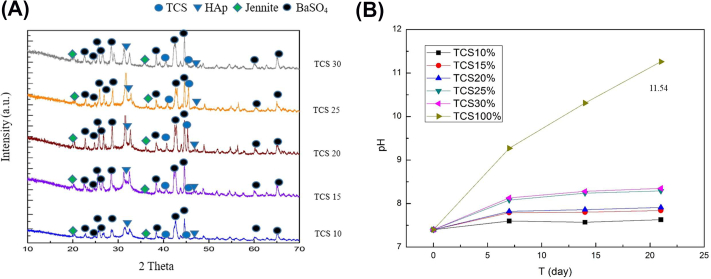
(A) The XRD patterns of 10–30% TCS in the PMMA/TCS system after soaking in SBF for seven days and (B) the pH values of the PMMA/TCS cements with different composition of TCS when immersed in the SBF for 21 days.

The components of our prepared PMMA/TCS cement were characterized using XRD. The diffraction patterns of the PMMA/TCS bone cement with a variation of TCS ratio (10%–30%) after immersing in SBF solution for seven days are shown in the Fig. 2(A). As shown, four distinct types of peaks were identified, including calcium silicate hydrate (CSH, Jennite) due to the hydration of TCS (Ca_3_SiO_5_) with Barium sulfate (BaSO_4_) added as a radiopaque agent in the commercial PMMA bone powder. In addition, hydroxyapatite (HAp) and un-hydrated TCS were also observed. Moreover, the characteristic peak of HAp at 2*θ* = 32.72° appeared in each hybrid combination with almost similar intensity. Therefore, the precipitation of HAp observed might be the main reason for the bioactivity of bone cement [24].

Fig. 2(B) demonstrates the pH values of the PMMA/TCS with different TCS composition when soaked in the SBF for 21 days. For 100% TCS, the higher pH change rate appeared in the first 7 days and it was about 28% from the initial pH value, followed by a gradual increase to 11.5 in 21 days. The formation of calcium hydroxide (Ca(OH)_2_) is accompanied by the tricalcium silicate hydration and causes an elevated pH value up to 12 [16,17].

A similar trend of pH variation was shown by the PMMA/TCS composite cement with a rapid pH increase in the first seven days but stabilized after that. At the same time, all the PMMA/TCS bone cement with different weight ratios of TCS (10%–30%) soaked in SBF showed a much lower pH than that of the pure TCS. Moreover, the stabilized pH values of composite cement increase as the weight percentage of the TCS in the PMMA increases, with the maximum pH value of 8.3 and 7.5 reached when the content of TCS is 30% and 10%, respectively, probably due to more of the Ca(OH)_2_ formed for the PMMA/TCS cement in the SBF environment. However, the pH values of living environments lower than 9.0 should be safe for the growth and proliferation of most human cells [38].

**Fig. 3:**
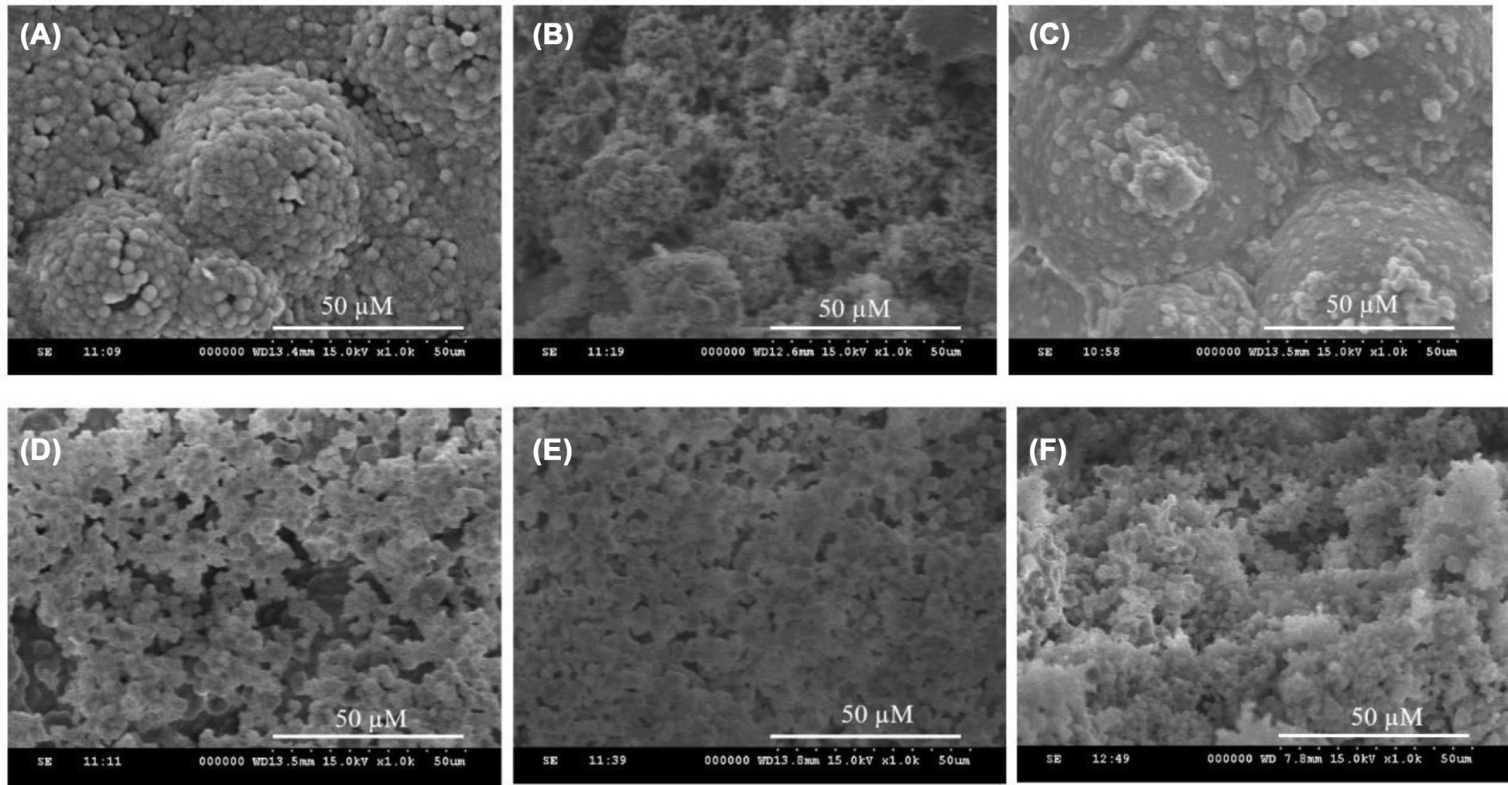
The SEM micrographs of PMMA/TCS cements with different weight ratios of TCS after soaking in SBF for 7 days. (A) PMMA; (B) TCS 10; (C) TCS 15_;_ (D) TCS 20_;_ (E) TCS 25_;_ (F) TCS 30_._

The SEM micrographs of PMMA/TCS cement surfaces are observed in Fig. 3. The surface of samples was covered by the crystalline HAp, with more of the apatite structures observed when the TCS content was increased (i.e., Fig. 3(A)–(F)). The HAp crystalline observed with higher magnification was flake-like and aggregated with typical bone-like apatite morphology.

Cement with PMMA alone is usually morphologically very dense, thus may not permit bone ingrowth. In contrast, TCS-based material may exhibit excellent i*n vitro* bioactivity when immersed in SBF due to the induction of surface HAp formation [39]. According to the XRD and SEM results, it proved that the PMMA/TCS composite cement could induce bone-like HAp structures within the cement in SBF, despite the composition, indicating the biological activity of the PMMA/TCS composite cement. Furthermore, from this apatite layer, the cement material could blend with surrounding bone tissues *in vivo* [40,41] through a chemical bonding to possess excellent bone-material compatibility [42]. Most importantly, the selected TCS content in this study will reinforce PMMA bone cement to confer the bioactivity without significantly reducing its mechanical properties.

**Fig. 4:**
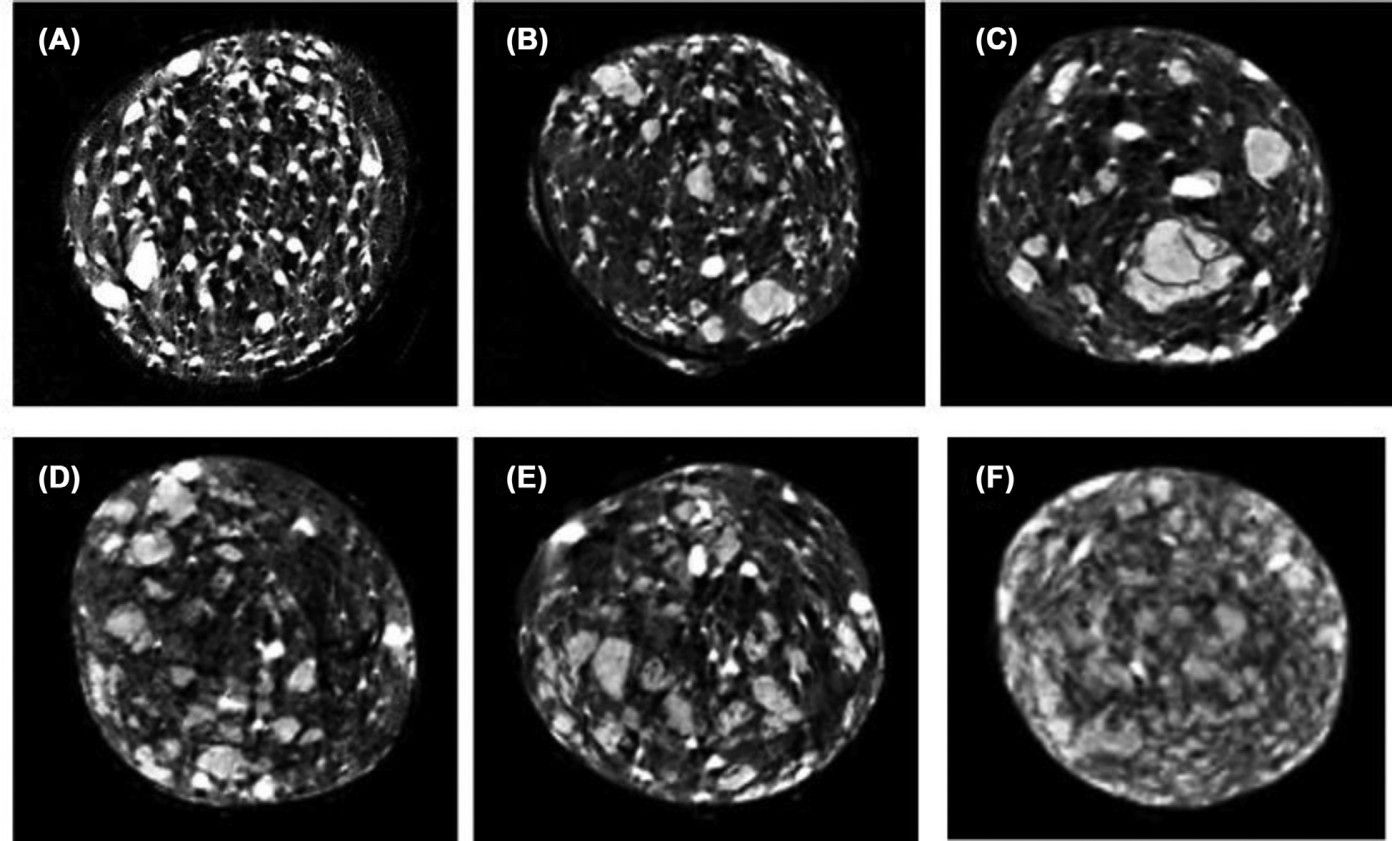
Micro-CT images of PMMA/TCS composite bone cement. (A) PMMA; (B) TCS 10; (C) TCS 15_;_ (D) TCS 20_;_ (E) TCS 25_;_ (F) TCS 30_._

Fig. 4 demonstrates the Micro-CT images of PMMA/TCS composite with bright white and grey areas corresponding to barium sulfate and TCS, respectively, within the dark PMMA matrix. Thus, we have the radiopaque properties of barium sulfate used for the enhanced contrast in PMMA. As shown, the TCS in the PMMA matrix was consistently dispersed, suggesting the excellent interfacial compatibility between TCS and PMMA. However, the particle size of TCS was uneven, and agglomeration happened partly when the TCS was increased in the PMMA matrix, which might be critical to the reduced compressive strength of the PMMA/TCS composite.

**Fig. 5:**
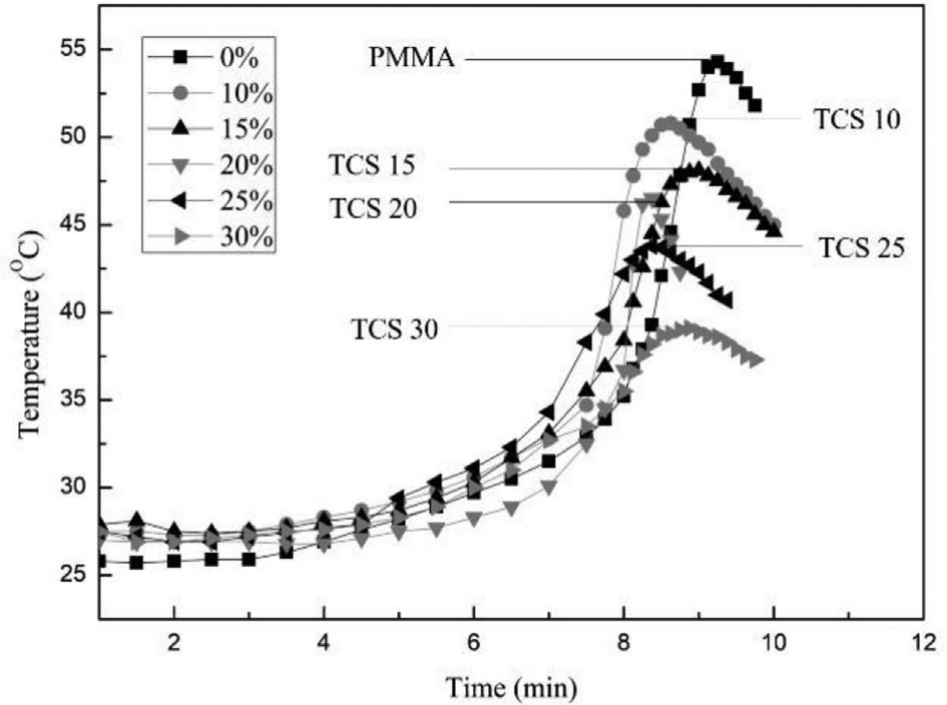
The exothermic temperature of pure PMMA and the PMMA/TCS bone cement with different weight ratios of TCS added.

Finally, the exothermic temperature of the cement samples was investigated in the temperature–time graph for change in temperature during the exothermic setting process of pure PMMA (0%) and PMMA/TCS bone cement composite samples with different TCS content involved (i.e., TCS weight ratios from 10 to 30% in a PMMA matrix). As shown in Fig. 5, when the reaction proceeds, the temperature reached the maximum, followed by a subsequent temperature decline. We can see that the maximum exothermic temperature produced by the pure PMMA is about 54 °C. The polymerization of MMA in the PMMA system categorizes high exothermic. The peak temperature attained is analogous to the quantity of heat generated due to the liquid phase polymerization reaction [43], which may cause necrosis in the living body. However, the peak temperature during the exothermic process tends to decrease, with the peak temperature reduced to 38 °C as the percentage of the TCS in PMMA bone cement increases to 30% (w/w). Thus, the insulated ceramic properties may slow the heat transfer release during the PMMA polymerization reaction [44]. Compared to pure PMMA cement, the reduced peak temperatures from the PMMA/TCS composite during the exothermic setting process would be much safer for the adjacent tissues during the in situ operations.

### Cell proliferation and ALP expression

**Fig. 6:**
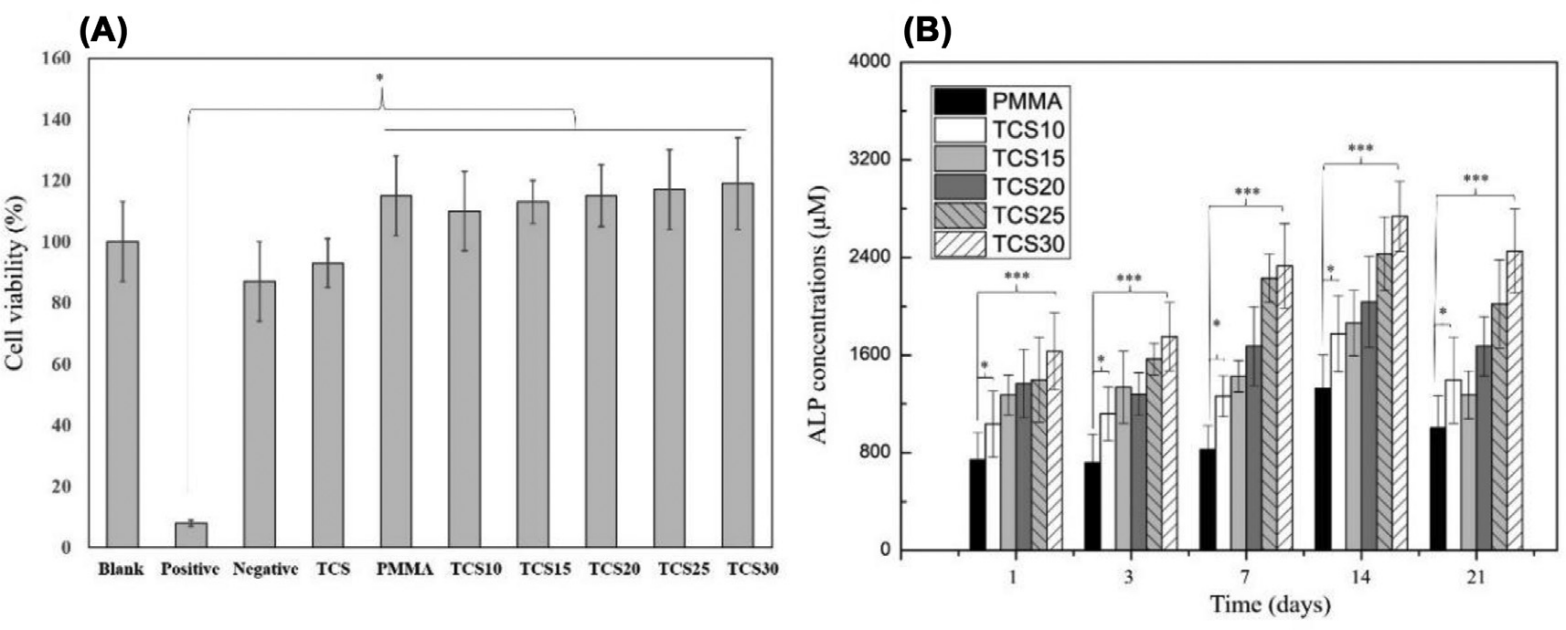
(A)The cell proliferation in dissolution extracts from PMMA/TCS composite with different TCS contents was analyzed using MTT assay. (B) Alkaline phosphate activity measurement of MG-63 cells seeded on the PMMA/TCS bone cement with different TCS contents for 1, 3, 7, 14, and 21 days. The error bars denote the mean ± SD for *n* = 5. ∗, *p* < 0.05; ∗∗∗, *p* < 0.001 were assessed as significant differences.

Fig. 6(A) exhibits the proliferation of the MG-63 cells on the PMMA/TCS composite surfaces with different variation of TCS composition. The cell proliferation was highly stimulated by the dissolution extracts of the pure PMMA and PMMA/TCS composite cement compared with the positive control (*p* < 0.05). The proliferation effect tends to increase with the increased TCS content, suggesting a positive impact of TCS on cell proliferation. Similar works were published that the cell proliferation and gene expression in osteoblast stimulated by the degradation of non-toxic ionic products from the calcium silicate bioactive glass [45,46]. However, the cell viability of pure TCS was smaller (*p* < 0.05) than the PMMA/TCS bone cement samples, suggesting that a specific TCS concentration range is required to stimulate cell proliferation from the dissolution products of PMMA/TCS composite. Thus, the effective TCS concentration in promoting cell proliferation is another critical topic for further investigation.

The secreted ALP concentrations from co-cultured MG63 with pure PMMA and PMMA/TCS bone cement with different weight ratios of TCS after 1, 3, 7, 14, and 21 days were shown in Fig. 6(B). ALP is a commonly known marker of bone formation and bone turnover which is expressed early in the bone development program, and is soon observed on the cell surface and in matrix vesicles. As shown in Fig. 6(B), the ALP concentration from MG-63 osteoblast-like cells on the different PMMA/TCS bone cement composition increased significantly (*p* < 0.05) after 1, 3, 7, 14, and 21 days in SBF. Also, at each measurement up to 21 days, the ALP concentration of the pure PMMA was significantly much lower (*p* < 0.001) than TCS 30. The decrease in the activity of ALP seen after 14 days may be due to the upregulation of other genes for example osteocalcin, which made the expression of ALP to decline. Also, it is known that the ALP helps in the mineralization of the bone cells and we can see that the activity of ALP is higher in the 14th day, which proves that the cells are completely mineralized and after that the cells started to differentiate [47]. Moreover, we assume that the release of calcium ions from TCS into culture medium started to deplete after 14 days, which obviously made the activity of ALP to decline at 21 days. Furthermore, when increasing the TCS in the PMMA bone cement, the corresponding ALP concentrations were also increased, suggesting an active ability of TCS to stimulate osteogenesis, which is critical for the formation of new bone.

Considering the results obtained from handling time, compressive strength, pH, exothermic temperature and cell viability experiments, the higher concentration of TCS (i.e., TCS 30) mixed with PMMA provided the optimal and acceptable values for choosing it as a safe and appropriate bone cement composition for testing against animal in the following I*n vivo* study.

### *In vivo* study analysis in animal

**Fig. 7:**
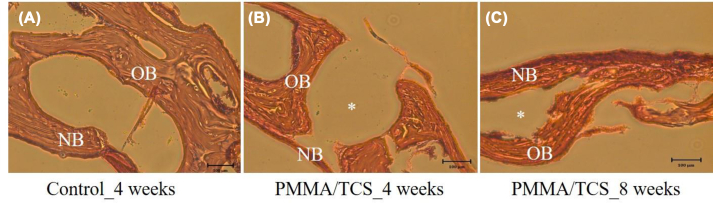
Representative histological images (stained with H&E) of the defect at fourth (B) and eighth (C) week post-implantation compared to the control group (A). The scale bar represents 100 μm (∗ denotes bone cement, N.B. denotes the new growth tissue after surgery, and O.B. indicates the extant bone before implantation).

*In vivo* study in rat calvarial defect repair model were used for evaluating the clinical application of the PMMA/TCS composite for bone regeneration. Representative histological H&E was shown in Fig. 7. As shown in Fig. 7(A), the microscopic image showed extensive new bone like tissue growth observed from the control groups four weeks after surgery. Only a thin layer of tissue was found around the PMMA/TCS cement four weeks after surgery (Fig. 7(B)). However, eight weeks after surgery, as shown in Fig. 7(C), bone like tissue growth at the bone-cement interface with a higher bone surface area was observed at the defect sites. These new growth tissues of the test group were similar to the control group, and the results didn't show significant inflammation. Thus, the *in vivo* results demonstrate the biocompatibility of the PMMA/TCS, especially at the interface and enhancement of bone like tissue growth after a more extended time. As a result, the TCS incorporated PMMA might improve the binding between in situ bone tissue and the bone cement, as TCS can facilitate strong osteocyte adhesion and differentiation. Furthermore, the unique bioactivity from this composite material design might provide much stable fixation after implantation.

Based on these results, the implanted PMMA/TCS bone cement with the bone-like apatite layer in the ambient fluids might enhance bone regeneration. Unfortunately, we also encountered some limitations in this study, which we wish to address for better insights. For example, the molecular mechanisms of how the bone cement interacts with the bone-forming cells are still unknown, and further investigation is needed. The reason behind the higher cell viability exerted by pure PMMA extracts is also inconclusive. Additionally, the use of radiopaque materials such as Barium sulfate, which lack X-ray absorption properties, could adversely affect the mechanical properties of the proposed PMMA/TCS bone cement composite. Furthermore, we are still unsure as to whether the proposed PMMA/TCS composite will meet the requirements for use in clinical studies in the near future. Further studies into the safety of the composite are required before clinical studies can begin.

## Conclusions

In this study, a novel PMMA-based composite cement fabricated from the conventional PMMA mixed with TCS with different weight ratio was successfully prepared. TCS was selected due to its low cost and excellent bioactivity properties. However, MMA has high mechanical strength as well as fast setting properties. Combining both PMMA/TCS characteristics and the impact of TCS on their integrated properties should have investigated.

The PMMA has rapid self-setting and high compressive strength but may cause thermal necrosis as the bone cement. Our developed bone cement suggests that TCS in the composite within the selected concentration range did not change the mechanical and handling characteristics of PMMA-based cement. On the other hand, the addition of TCS could induce bone-like apatite formation in SBF, making the composite cement bioactive. The apatite layer may further insulate the heat transfer and reduce the peak temperature observed during the cement setting process. Also, the dissolution extracts of composite could stimulate cell proliferation within the selected TCS concentration range. The *in vivo* animal study further demonstrates that the PMMA/TCS bone cement could enhance bone regeneration. Thus the novel PMMA based composite cement with the addition of TCS in the selected concentration range exhibiting the stabilized pH and the exothermic temperature values might be a potential bone filler for future orthopedic surgeries.

## Conflicts of interest

The authors have declared that no conflicts of interest exists.
