# Supplementary material for: Study of polymethylmethacrylate/tricalcium silicate composite cement for orthopedic application
Source: Biomed J. 2022 May 29;46(3):100540. doi: 10.1016/j.bj.2022.05.005 (PMC10209682; doi:10.1016/j.bj.2022.05.005)
Supplement: Multimedia component 1 [file mmc1.docx]

**Study of polymethylmethacrylate/tricalcium silicate composite cement for orthopedic application**

*Yang Wei ^1^, Nareshkumar Baskaran ^1^, Huey-Yuan Wang ^2^, Yu-Chieh Su ^1^, Sasza Chyntara Nabilla ^3^, Ren-Jei Chung ^1,^**

^1^ Department of Chemical Engineering and Biotechnology, National Taipei University of Technology (Taipei Tech), Taipei, Taiwan

^2^ Department of Stomatology, MacKay Memorial Hospital, Taipei, Taiwan

^3^ Department of Materials, University of Oxford, Oxford, United Kingdom

This supporting information contains (*i*) Preparation of raw materials, (*ii*) Simulated body fluid (SBF), (*iii*) Cell culture medium, *(iv)* MTT assay *(v)* In vivo study and *(vi)* Handling time of Pure TCS with the addition of different concentration of citric acid.

**S1.** **Preparation of raw materials**

PMMA (MW=30K) powder was purchased from Lucite International Ltd., Billingham, UK. The end configuration of the cement referred to CMW-1, a commercial product from DePuy company, Blackpool England, and the composition of the cement powder includes 88 wt.% PMMA powder, 10% barium sulfate, and two wt.% benzoyl peroxide. The liquid part was composed of 98.5 wt.% Methyl Methacrylate (MMA), 1.5 wt.% N, N-Dimethyl-p-toluidine, and 75 ppm hydroquinone. The content of TCS as an additive to the powder part was from 0 wt.% (TCS) to 80 wt.% (TCS 80).

From the sol-gel method [1], tricalcium silicate (TCS) powder was synthesized by mixing the raw materials including Ca(NO_3_)_2_·4H_2_O and Si(OC_2_H_5_)_4_ (TEOS) along with a catalyst (Nitric acid, HNO_3_). In brief, 1M TEOS was mixed with 10 M of ethanol under constant stirring with the subsequent addition of 3 M Ca(NO_3_)_2_·4H_2_O (calcium precursor). The resulting solution was dried for 24 hours at 80 °C and was sieved 400-mesh after calcination at 1450 °C for 14 hours.

**S2. Simulated body fluid (SBF)**

According to Kokubo [2], an ion concentration of SBF solution nearly equals human blood plasma. The cement samples (diameter=10 mm, height=5 mm) were immersed in SBF at 37°C in a 100% humidity water bath and were stirred at 100 rpm for three weeks. The surface area to volume ratio was 0.1 cm^−1^ [3]. Finally, the sample columns were rinsed using DI water and were dried at 24 °C for further characterizations.

**S3. MTT assay**

First, the samples were wholly sterilized under ultraviolet light for 3 hours. Then the bone cement was soaked in phosphate-buffered saline (PBS, Sigma-Aldrich, USA) solution and was washed thoroughly. Finally, the MG-63 cells were seeded in a 96-well plate at densities of 5 × 10^4^ cells/well and were incubated for 24 hours. From which, the complete growth culture medium was considered as a blank. In contrast, cells cultured with 1% triton in medium and Teflon molds were regarded as a positive and negative control, respectively. We have the extract solutions prepared by immersing the completely sterilized bone cement samples in a DMEM with 1% P.S., and 10% heat-inactivated FBS, followed by placing them in an incubator maintained at 37 °C. The protocol outlined in the ISO 10993 standard was used to prepare the extracted solution [4]. The prepared PMMA/TCS extracting solution was filtered with single-use sterilized filters (0.20 µm) (DISMIC, Japan), and the solution was experienced 24-hour incubation at 37°C, 5% CO_2_. Then, it was added to the 96-well plate with MG-63 cells and kept undisturbed in the incubator for three days. The 20 *μ*l of the MTT reagent was added to the plate and later dissolved by adding dimethyl sulfoxide (DMSO) solvent (Sigma Aldrich, UK) to form a dark blue formazan. The optical density (O.D.) was measured at 595 nm using an ELISA plate reader (TECAN Sunrise, USA). The number of living was determined using the absorbance values that consider being proportional to each other [5].

**S4. *In vivo* study**

Chemicals mentioned in this *in vivo* study were of analytical grade and were purchased from Sigma-Aldrich (Saint Louis, USA) unless otherwise stated. The Sprague-Dawley (S.D.) rats model weighed about 300 g obtained from the National Laboratory Animal Center, Taiwan. Before experimentation, all the rats should have a minimum one-week acclimation and housed adequately with access to the standard rat food and periodically fed with water. Rats were first anesthetized with 0.3 mL of Zoletil and Rompun at the ratio of 1:2, and then we have their top heads shaved to expose the skin. A 1-cm sagittal skin incision was made through the midline of the head using a #10 scalpel, followed by the exposure of calvarium through full-thickness skin and periosteum retraction. After the frontal bone exposure, a bone defect with a diameter of 6 mm was made using a sterile electric drill, followed by the placement of 5-mm diameter, 1-mm thick implant cement disc (TCS 30) over the defect site. Wounds were then sutured, cleaned with 2% iodine, and closed with 4-0 nylon thread. After the fourth and eighth week of surgery, all the experimental rats were euthanized by injecting 90-120 mg of sodium salt (Pentobarbital Sodium) per kg body weight through intraperitoneal injection (IP). Finally, the skull was further isolated with the extracted bone tissue in 10% EDTA, dehydrated in alcohol, and fixed with paraformaldehyde for 24 h at 4°C. We have the bone cement disc gently removed after that. After the sliced serial cross-sections, we have each fixed bony specimen assigned to the paraffin slice for hematoxylin-eosin (HE) staining.

**S5. Handling time of Pure TCS with the addition of different concentration of citric acid**

From the figure, we can see that when the concentration of citric acid increased, both the working and setting time of TCS bone cement decreased significantly, reaching almost 2-3 mins when the concentration is 5 molar (M), which is far below the acceptable range (10- 15 minutes). However, studies have also shown that increasing the concentration of citric acid in the TCS bone cement had detrimental effect on its mechanical property, especially compressive strength. Thus, considering the curing time and mechanical strength into account, we have chosen the minimal concentration of citric acid (i.e., 0.5 M).

**
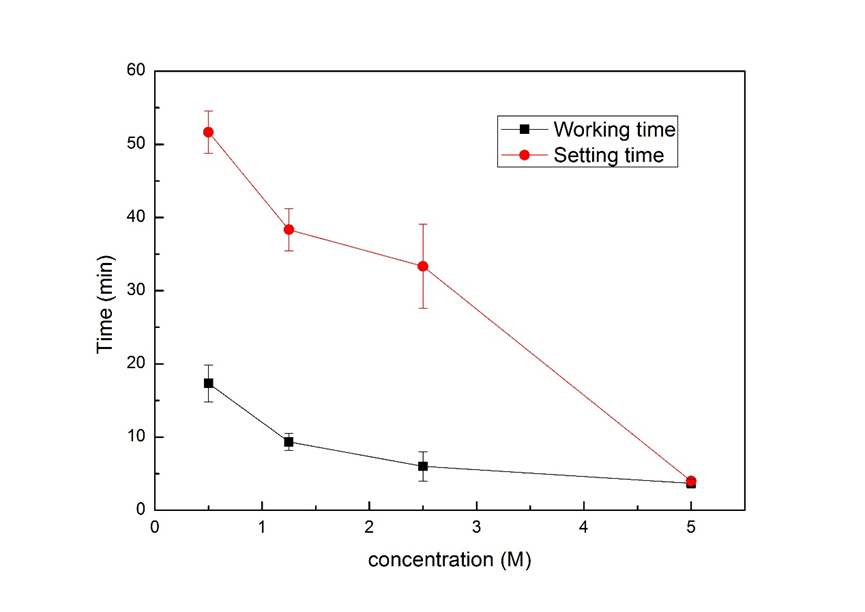
**

**Figure S1.** Setting and working time of TCS bone cement with the addition of different concentration of citric acid

**References:**

[1] Zhao W, Chang J. Sol–gel synthesis and in vitro bioactivity of tricalcium silicate powders. Mater. Lett 2004;58:2350-3.

[2] Kokubo T. Surface chemistry of bioactive glass-ceramics. J. Non-Cryst. Solids X 1990;120:138-51.

[3] Huan Z, Chang J. Novel tricalcium silicate/monocalcium phosphate monohydrate composite bone cement. J Biomed Mater Res B Appl Biomater 2007;82:352-9.

[4] Iso E. Biological evaluation of medical devices-Part 5: Tests for cytotoxicity: in vitro methods. German version EN ISO 1999:10993-5.

[5] O'Hare P, Meenan BJ, Burke GA, Byrne G, Dowling D, Hunt JA, et al. Biological responses to hydroxyapatite surfaces deposited via a co-incident microblasting technique. Biomaterials 2010;31:515-22.
